# Unusual combination of lesions of the traumatic hand: closed central slip laceration of the extensor and interphalangeal thumb joint's dislocation (a case report)

**DOI:** 10.11604/pamj.2014.18.230.3240

**Published:** 2014-07-18

**Authors:** Hassan Boussakri, Mohamad Azarkane, Omar Dahmani, Mohamad Elidrissi, Mohamed Shimi, Abdelhalim Elibrahimi, Abdelmajid Elmrini

**Affiliations:** 1 Department of Orthopaedic Surgery ( B4),CHU Hassan II Hospital, University of Sidi Mohammed Ben Abdellah, 3000 Fez, Morocco

**Keywords:** Traumatic hand, boutonniere deformity, thumb dislocation, pincer grasp

## Abstract

From the functional standpoint, the hand is one of the most important organs of the body. However, its significance depends largely upon the pincer action of the thumb-index. The management of traumatic lesions of the hand is nowadays’ subject of numerous scientific discussions. We present here the case of a patient with a recent laceration of the central slip of the extensor tendon with boutonniere deformity linked to a dislocated interphalangeal thumb of the same hand with a loss of force of the clip thumb and index finger. This combination is a rare lesional of the traumatic hand that has not been previously reported in any orthopedic literature. It was observed after adopting the orthopedic treatment that the range of motion of its joint was at the same level as its healthy side without observing any redislocations during the 6-month follow-up period.

## Introduction

To pick up an object we using the hand, this function provided particularly by thumb-index pinch [1]. This operation of the clip is provided by the digital and the muscle with the tendon bone chain termination. Moreover, the extensor apparatus of the finger is a complex structure and injury can lead to significant digital dysfunction. Besides, closed central slip injuries could be missed and diagnosis might be delayed because of the lack of an open wound and often with no radiographic abnormality; this could results boutonniere deformities if they were untreated [2]. Many procedures were described in literature with no recommendation of a standard treatment (whether Conservative treatment or surgical) [3]. Dorsal dislocations of the interphalangeal joint are common injuries and reduction is usually obtained by manipulation. When it is irreducible then we have to think of an interposition between the joint surfaces. [4] In this report we aim to present the case of a patient with laceration of the central slip of the extensor tendon at the proximal interphalangeal (PIP) joint with volar displacement of the lateral bands which could results the so-called boutonniere deformity that includes a loss of extension at the PIP joint and compensatory hyperextension of the distal interphalangeal (DIP) joint associated to a dislocated interphalangeal thumb of the same hand with less power in the clip thumb and index finger.

## Patient and observation

**Figure 1:**
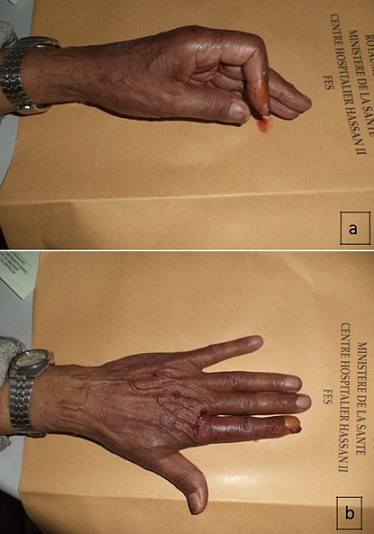
The photograph shows boutonniere deformity of the index evoking rupture of the central slip with deformity of the left thumb in favor of dislocation of the interphalangeal joints

**Figure 2:**
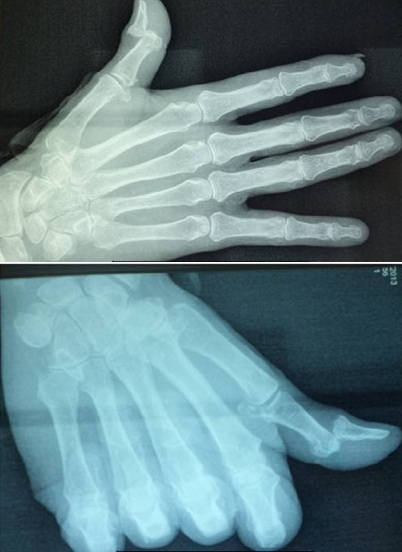
Anteroposterior and lateral radiographs display the dorsal dislocations of the interphalangeal joint

**Figure 3:**
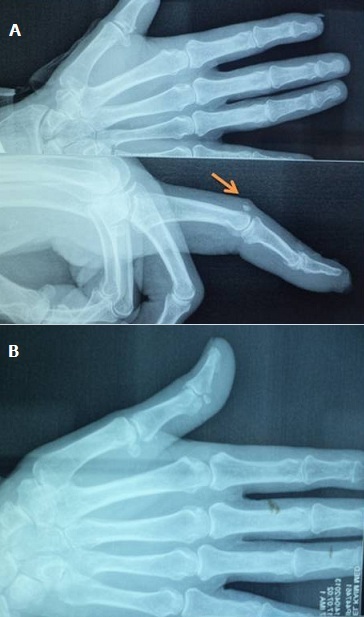
A) avulsion Central Slip of the index; B) radiological control objectifying reduction of the dislocation of the interphalangeal joint of the thumb

**Figure 4:**
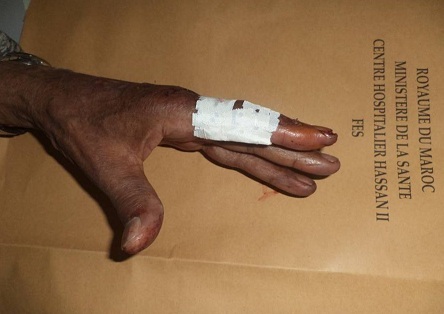
The photograph shows orthopedic treatment with immobilization of avulsion Central Slip of the index

**Figure 5:**
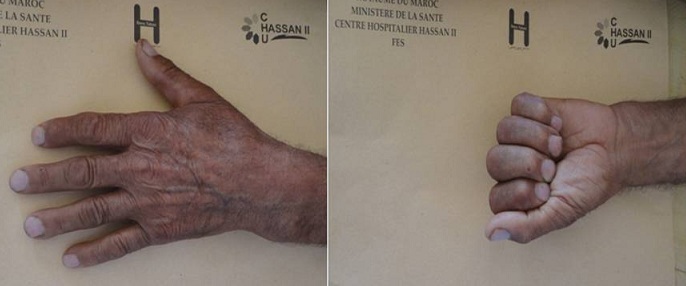
The clinical results were satisfactory after a mean of 6 months

**Figure 6:**
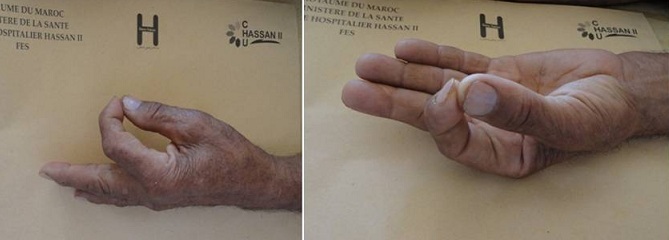
The clinical results were satisfactory after a mean of 6 months

A 60 -year-old man, who is a right-hand dominant, was seen at the emergency department of our hospital (CHU Hassan II Fez, Morocco). The patient fell on his left hand from a height of approximately 1 meter after losing control on his bicycle. The patient tried to support his elbow of the injured side with the other arm, but at the same time he was complaining about so much pain, being unable to move his left hand (thumb and index finger). A physical examination identified a slight swelling and limitation of movement of the hand (index and thumb). The patient was unable to perform movements with his left hand which did not allow passive movements. As a result, he has got a deformed thumb with a deformity of index boutonniere. Neurovascular examination of the digit was normal (Figure 1). Nonetheless, his radiography showed an interphalangeal dislocation thumb with Closed Central Slip avulsion of the index finger (Figure 2, Figure 3 (e)). Additionally, a closed reduction of the interphalangienne dislocation of the thumb was performed in the emergency department using a digital block. The Neurovascular exam after reduction was also normal. The post-reduction X-rays obtained in the emergency room showed a restoration of articular congruity at both joints (Figure 3 (f)). This is why; we adopted an orthopedic treatment with immobilization (Figure 4), and physiotherapy to restore passive extension and flexion of the proximal interphalangeal joint. It was observed that the range of motion of its joint was at the same level as its healthy side without any redislocations observed during the 6-month follow-up period and the patient made good progress. (Figure 5, Figure 6).

## Discussion

From the functional standpoint, the hand is one of the most important organs of the body. However, its significance depends largely upon the pincer action of the thumb-index. The extreme mobility of the first metacarpal bone made the thumb functionally as important as all the remaining fingers. Due to the high functional salience even the slightest degree of impairment may not be acceptable [3]. Through the size, weight, shape of the object and method gripping, we could define several types of plugs among which are the bidigitales taken. The later, are divided into two categories: terminopulpar clamps (pinch) and pulpo-side (key grip) [5]. The operation of this clip is provided by the digital and the muscle with the tendon bone chain termination and the grip force is assured essentially by thumb-index pinch [6]. A very scanty body of literature is available on the subject with no similar cases or documents were previously reported neither published. Dorsal dislocations of the interphalangeal joint are common injuries, and reduction is usually obtained by manipulation. When it is irreducible then we have to think of an interposition between the joint surfaces [4]. Closed Zone III or “boutonniere” injuries are managed conservatively unless there is evidence of displaced avulsion fractures at the base of the middle phalanx, axial and lateral instability of the PIP associated with loss of active Or passive extension of the joint or of a failed non-operative treatment [7]. Disruption or laceration of the central slip of the extensor tendon at the proximal interphalangeal (PIP) joint with volar displacement of the lateral bands can result the so-called boutonniere deformity which includes loss of extension at the PIP joint and compensatory hyperextension of the distal interphalangeal (DIP) joint. Many procedures have been described in the literature with no recommendation of a standard treatment [8]. The section or rupture of the median strip of the extensor is the result of the absence of adequate chronic boutonniere deformity treatment [8]. The injury is only diagnosed at this stage and appropriate splinting and hand therapy instituted. A PIPJ splintage and distal interphalangeal joint,(DIPJ) flexion exercise regime is the mainstay of treatment for closed central slip rupture. However, in established boutonniere deformities that are no longer correctable, surgical release of the PIPJ and reconstruction of the central slip might be the only intervention available to the patient [9, 10]. Actually, diagnosed closed central slip injuries could be treated with extension splinting of the PIPJ - combined with maximum forced active flexion exercises of the DIPJ in order to restore the normal tendon balance and precise length relationship of the central slip and lateral bands. Another option is the immediate surgical repair of central slip injuries including reattachment of the avulsed central slip using a commercially available bone anchor, and reconstruction of the damaged central slip with adjacent lateral band tissues [10, 11].

## Conclusion

The interphalangeal thumb joint's dislocations are commonly encountered injuries in the traumatic hand, less common Closed Central Slip avulsion of the index finger. This combination is a rare lesional of the traumatic hand which was not previously reported in the orthopedic literature. The orthopedic treatment usually provides satisfactory functional results.
